# Response of the eukaryotic plankton community to the cyanobacterial biomass cycle over 6 years in two subtropical reservoirs

**DOI:** 10.1038/s41396-019-0417-9

**Published:** 2019-05-03

**Authors:** Lemian Liu, Huihuang Chen, Min Liu, Jun R. Yang, Peng Xiao, David M. Wilkinson, Jun Yang

**Affiliations:** 1 Aquatic EcoHealth Group, Key Laboratory of Urban Environment and Health, Institute of Urban Environment, Chinese Academy of Sciences, 361021 Xiamen, China; 2 Technical Innovation Service Platform for High Value and High Quality Utilization of Marine Organism, Fuzhou University, 350108 Fuzhou, China; 3 University of Chinese Academy of Sciences, 100049 Beijing, China; 4 School of Life Sciences, University of Lincoln, Lincoln, LN6 7TS UK

**Keywords:** Microbial ecology, Community ecology, Freshwater ecology

## Abstract

Although it is widely recognized that cyanobacterial blooms have substantial influence on the plankton community in general, their correlations with the whole community of eukaryotic plankton at longer time scales remain largely unknown. Here, we investigated the temporal dynamics of eukaryotic plankton communities in two subtropical reservoirs over a 6-year period (2010–2015) following one cyanobacterial biomass cycle—the cyanobacterial bloom (middle 2010), cyanobacteria decrease (late 2010–early 2011), non-bloom (2011–2014), cyanobacteria increase, and second bloom (late 2014–2015). The eukaryotic community succession that strongly correlated with this cyanobacterial biomass cycle was divided into four periods, and each period had distinct characteristics in cyanobacterial biomass and environments in both reservoirs. Integrated co-occurrence networks of eukaryotic plankton based on the whole study period revealed that the cyanobacterial biomass had remarkably high network centralities, and the eukaryotic OTUs that had stronger correlations with the cyanobacterial biomass exhibited higher centralities. The integrated networks were also modularly responded to different eukaryotic succession periods, and therefore correlated with the cyanobacterial biomass cycle. Moreover, sub-networks based on the different eukaryotic succession periods indicated that the eukaryotic co-occurrence patterns were not constant but varied largely associating with the cyanobacterial biomass. Based on these long-term observations, our results reveal that the cyanobacterial biomass cycle created distinct niches between persistent bloom, non-bloom, decrease and increase of cyanobacteria, and therefore associated with distinct eukaryotic plankton patterns. Our results have important implications for understanding how complex aquatic plankton communities respond to cyanobacterial blooms under the changing environments.

## Introduction

In freshwater ecosystems, cyanobacterial blooms are increasingly common around the world as a result of eutrophication and global warming [1]. Cyanobacterial blooms can produce toxins in water, posing a potential risk to humans and domestic animals [2]. They are also associated with shifts in eukaryotic plankton (in this study, defined as planktonic algae, protozoa, fungi, and small metazoa), which are important components of freshwater ecosystems [3–5]. There are a number of potential mechanisms at multi-level and multi-aspect for these shifts. First, the production of toxic metabolites, change of environmental conditions, and competition from cyanobacterial blooms can cause negative effects on eukaryotic plankton [4, 6–8]. Second, the cyanobacteria aggregation is composed of inedible colonies or filaments that are too large to be ingested by most zooplankton [9]. Third, heterotrophic protists and metazoan zooplankton can have mixed effects, either preventing cyanobacteria blooms via grazing on cyanobacteria [10] or promoting cyanobacterial blooms via grazing on other phytoplankton [11]. Fourth, cyanobacterial blooms can indirectly influence the heterotrophic protists through effects on heterotrophic bacteria and surrounding environments [12–14]. Despite cyanobacterial blooms being strongly correlated with shifts of the whole eukaryotic plankton communities, to our best knowledge, studies focused on wide cross-kingdom investigations are very few, mostly from marine ecosystems (e.g., diatom bloom), and generally based on results from multiple sampling surveys and different projects [4, 15, 16].

In eutrophic waters, cyanobacterial biomass can cyclically change from bloom to non-bloom states over a series of years, resulting in associated microbial community changes [17]. In this study, we followed one cyanobacterial biomass cycle over a 6-year period (2010–2015) in two subtropical reservoirs (mainly dominated by *Cylindrospermopsis raciborskii*)—the cyanobacterial persistent bloom (middle 2010), rapid decrease (late 2010–early 2011), non-bloom (2011–2014), increase, and second bloom (late 2014–2015). This cyanobacterial cycle included a remarkable variation of cyanobacterial biomass and might associate with different characteristics between cyanobacterial bloom, non-bloom, increase, and decrease phases [10, 16]. Therefore, we hypothesized that the cyanobacterial biomass cycle inevitably associated with several distinct patterns in eukaryotic plankton community composition and co-occurrence. However, until now, the longer-term dynamics (in this study, 6 years) of eukaryotic plankton community composition and co-occurrence patterns associated with the cyanobacterial biomass cycle particularly in reservoir ecosystems have not been investigated.

Ecological communities are composed of a variety species, with multiple interactions between them. These interactions can markedly determine the species occurrence patterns [18]. Recently, an increasing number of studies have used network analysis to examine the microbe co-occurrences and the environmental conditions (including cyanobacterial bloom) that correlate with these co-occurrences [15, 19, 20]. A network represents a set of nodes connected by directed or undirected edges—where the nodes are a component of the network and are joined by an edge if they potentially interact. This area of mathematics has wide applicability, for example to interacting neurons, businesses, or the internet [21]. Interpreted ecologically, the nodes represent species (or environmental factors) and edges represent associations between nodes [18]. To date, most previous studies have treated the microbial co-occurrence network as a static system. Previous studies have shown that the co-occurrence between the large organisms are not static but varied as the environmental changes over time [22, 23]. The local environment strongly affects classic ecological processes such as the intrinsic growth rate and niche breadth, so that species existence, persistence, and the consequence of co-occurrence patterns will vary along environmental gradients [24, 25]. Another mechanism is that a network can have multi-functional agents that are similar in some, but not all, functions providing some redundancy [26]. In such a system, the microbial network potentially provides a buffer against disturbance, as alternative pathways with different combinations of agents can be recruited to fulfill specific functions [27]. Both mechanisms suggest possible reasons for co-occurrence network changing with environmental variation. The cyanobacterial biomass cycle is accompanied by a remarkable change in environmental conditions [10, 16], and therefore inevitably and obviously associate with a varied eukaryotic plankton co-occurrence pattern along this environmental gradient.

How plankton communities respond to cyanobacterial change is very complicated. In this study, we used long-term data on both eukaryotic plankton communities and cyanobacterial biomass to explore how cyanobacterial biomass changes might affect eukaryotic plankton community in two adjacent subtropical reservoirs in southeast China over a 6-year period associated with one cyanobacterial biomass cycle. We aimed to determine: (1) the response characteristics of plankton community composition and diversity to the cyanobacterial biomass cycle; (2) how the cyanobacterial biomass cycle associates with the eukaryotic plankton co-occurrence patterns.

## Materials and methods

### Study sites, sampling, and physical and chemical factors

The two reservoirs studied, Shidou Reservoir and Bantou Reservoir, are located in Xiamen, southeast China (Fig. S1). Xiamen has a subtropical monsoon climate, characterized by long, humid, and hot summers and short, dry, and mild winters. The annual mean temperature is 20.7 °C and the annual mean precipitation is 1335.8 mm in Xiamen. Shidou Reservoir is a large deep reservoir (total storage capacity 61.4 million m^3^ with a mean water depth over the three sampling stations of 13.9 m) within a wooded catchment, while Bantou is a smaller and shallow reservoir (total storage capacity 4.4 million m^3^, mean water depth 7.2 m) with a similar catchment immediately downstream of Shidou Reservoir. There is a dam between Shidou and Bantou reservoirs, and water flows into Bantou from Shidou when the water level is high in Shidou. Details of these two reservoirs were described in our previous studies [28, 29].

There were 30 sampling visits from May 2010 to October 2015 for Shidou and Bantou reservoirs, environmental  details are given in Fig. S2. On each visit, three replicate samples were taken from each reservoir (near the inflow, near the outflow, and the middle of each reservoir) (Fig. S1). A total of 90 water samples were collected from the surface water (upper 50 cm) for each reservoir. About 500 mL water was pre-filtered through a 200 μm mesh to remove large metazoans and other particles, then filtered using 0.2 μm pore-size polycarbonate membrane (47 mm diameter, Millipore, Billerica, MA, USA) to collect the plankton cells.

Water temperature (Temp), electrical conductivity (EC), pH, and dissolved oxygen (DO), were measured in situ with a Hydrolab DS5 multi-parameter water quality analyzer (Hach, Loveland, CO, USA). Water transparency (Trans) was measured with a Secchi disk. Total nitrogen (TN), ammonium nitrogen (NH_4_-N), nitrite and nitrate nitrogen (NO_x_-N), and total phosphorus (TP) were analyzed according to standard methods [30].

### DNA extraction, PCR, and Illumina sequencing

Total DNA of eukaryotic plankton communities was extracted directly from the membrane using the FastDNA SPIN Kit and the FastPrep Instrument (MP Biomedicals, Santa Ana, CA, USA) according to the manufacturer’s instructions. The primer pair 1380F and 1510R [31] with barcodes were used to amplify the hypervariable V9 region of the eukaryotic 18S rRNA gene. PCR reaction contained 15 μL of Phusion High-Fidelity PCR Master Mix (New England Biolabs, Beverly, MA, USA), 0.2 μM of each primer, and 10 ng of target DNA. The reactions included an initial denaturation at 98 °C for 1 min, followed by 30 cycles of 10 s at 98 °C, 30 s at 50 °C, and 60 s at 72 °C. At the end of the amplification, the amplicons were subjected to final 10 min extension at 72 °C. PCR products from triplicate reactions per sample were pooled and gel-purified. In total, 180 libraries were sequenced on the Illumina HiSeq platform (Illumina Inc., San Diego, CA, USA) using a paired-end strategy.

### Bioinformatics

Paired-end Illumina V9 region of 18S rRNA gene sequences was processed using Vsearch 1.9.1 [32]. Singletons and likely chimeras were also discarded using default settings in Vsearch. Quality filtered reads were assigned to OTUs at a 97% sequence similarity threshold. Representative sequences from each OTU were identified by the Protist Ribosomal Reference (PR2) database [33]. Unassigned (sequence similarity to a reference sequence is <80%) and OTUs with <8 reads were removed before the downstream analyses. For our data analyses, we randomly selected a subset of 29057 reads at 97% threshold from each sample to standardize sequencing effort. The final total data set retained 5,230,260 reads at 97% sequence similarity level. All sequence data from this study have been deposited in the public NCBI Sequence Read Archive (SRA) database under the BioProject number PRJNA415265 and the accession number SRP121028.

The definition of abundant and rare OTUs is depended on the relative abundance following a recent study [4]. The OTUs were divided into four main categories: abundant taxa—relative abundance ≥1% in a sample but never <0.01% in all samples; rare taxa—relative abundance <0.01% in a sample, and never >1% in all samples; conditionally rare and abundant taxa—relative abundance ≥1% in some samples and <0.01% in other samples; moderate taxa—relative abundance between 0.01 and 1% in all samples.

### Microscopy analysis of cyanobacteria and total algae

For the analysis of total algae (including eukaryotic algae and cyanobacteria), a total of 2.5 L of surface water samples were fixed in situ with 1% Lugol’s iodine solution and were concentrated to a final volume of 50 mL [34]. Algae were identified and counted using an inverted microscope (Motic, Xiamen, China) following Shen et al. [35], Zhang and Huang [36], and Hu and Wei [37]. A total of three subsamples were investigated for each sample, and at least 500 individuals were identified and counted for each sample. The abundance for each algal species was transformed to biomass following Hillebrand et al. [38]. We use two measures of cyanobacterial biomass in this study—absolute biomass (mg/L) and relative biomass (%) (i.e., the ratio of cyanobacterial biomass to total algal biomass identified by microscopy).

### Definition of cyanobacterial bloom

Although numerous references mentioned the mass occurrence of cyanobacteria, there is no universal definition of a “bloom”; typically a bloom is informally defined as a growth dense enough to color the surface waters [39, 40]. Given that both the relative and absolute biomass is crucial for definition of “bloom”, we use the term “bloom” for conditions when cyanobacteria are >50% (dominance) [41] of total algal biomass and cyanobacterial biomass is >10 mg/L. This represented a moderate probability of adverse health effects of cyanobacterial bloom according to the World Health Organization’s guideline, which has subsequently been adopted by other studies [42, 43].

### Analysis of plankton community composition

Non-metric multidimensional scaling (NMDS) ordination and analysis of similarities (ANOSIM) were used to investigate differences in eukaryotic plankton community composition among groups. The community composition between samples were analyzed using the Bray–Curtis similarity of eukaryotic OTU reads relative abundance [44]. These analyses were run in PRIMER 7.0. We used Mantel test (vegan package in R) [45] to investigate the correlations between Euclidean distance of environmental factors and Bray–Curtis dissimilarity of eukaryotic plankton communities.

### Structural equation model

Direct dependencies between the response variables (NMDS axes 1 and 2) and all groups of relevant physical and chemical factors (water temperature, electric conductivity, pH, dissolved oxygen, transparency, TN, TP, NO_x_-N, and NH_4_-N) as well as cyanobacterial biomass were assessed in a structural equation model using path analysis [46, 47]. We started with initial models that included all plausible pathways between eukaryotic plankton communities (NMDS axes 1 and 2), relevant physical, chemical and cyanobacterial biomass factors except the relationships between the physical and chemical factors. To reduce the complexity of the structural equation models, the correlations between the physical/chemical factors were not included in the initial models. Subsequently, the significance of each path-coefficient was tested by its critical ratio (*P* < 0.05), and non-significant paths were removed in a stepwise fashion until all remaining paths were significant [48]. The overall fit of the final model was evaluated with the goodness-of-fit index (GFI), Bentler comparative fit index (CFI), and Chi-square test, respectively [48]. The structural equation analysis was performed using the software package AMOS version 19 (IBM Corp., Armonk, NY, USA).

### Network construction

**Fig. 1:**
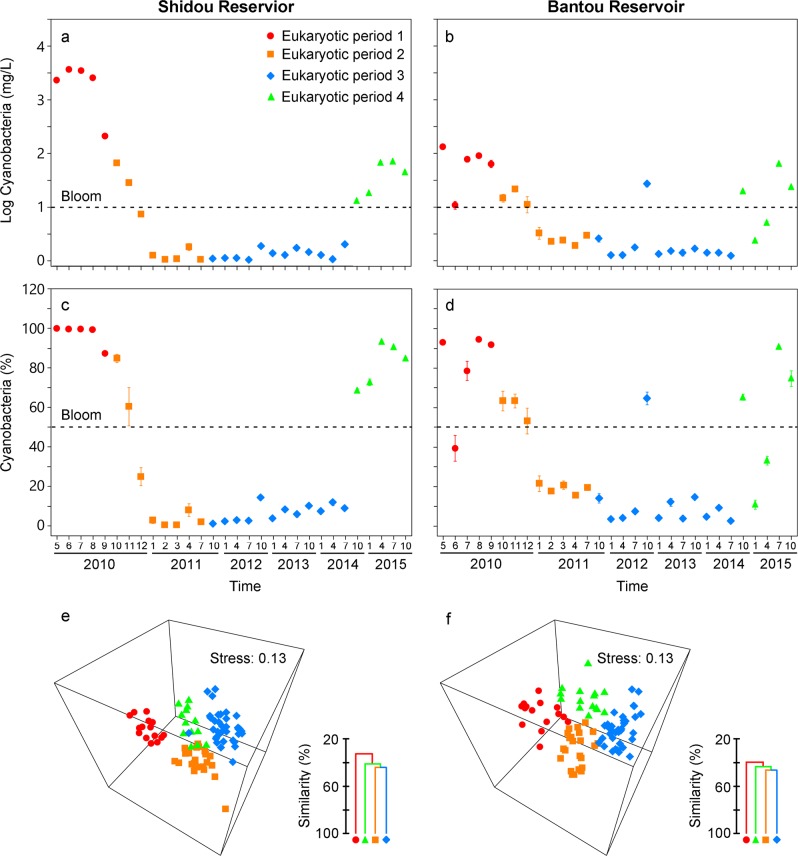
Synchronous dynamics and correlations between microeukaryotic plankton and cyanobacterial biomass over 6 years in two subtropical reservoirs. **a**, **b** Variation of the log cyanobacterial absolute biomass (mg/L). **c**, **d** Variation of the percentage of cyanobacterial biomass to total algal biomass (%). **e**, **f** Non-metric multidimensional scaling (NMDS) ordination and clustering showing the inter-annual variation of eukaryotic plankton communities was corresponding to cyanobacterial dynamics in Shidou and Bantou reservoirs. Four distinct periods were defined based on plankton communities and environmental variables

We constructed one integrated network based on samples from over the whole study period (May 2010–Oct 2015, 90 samples for each network) for each reservoir. We also constructed four sub-networks for each reservoir based on samples in the four succession periods of eukaryotic plankton community, respectively (see Fig. 1 for the four eukaryotic succession periods).

To reduce noise and thus false-positive predictions, for each network, we restricted our analysis to taxa presence in >1/3 of the samples. All eleven environmental factors (water temperature, electric conductivity, pH, dissolved oxygen, transparency, total nitrogen, NH_4_-N, NO_x_-N, total phosphorus, cyanobacterial absolute biomass, and relative biomass) were included in the two integrated networks.

We used SparCC to explore the relationships that were consistent among the eukaryotic plankton taxa and environmental factors [49]. Only robust (|r| > 0.4) and statistically significant (*P*-value < 0.01) correlations were incorporated into network analyses. To correct the unequal sampling efforts between the different eukaryotic succession periods, for the sub-networks in different eukaryotic succession periods, only the top 10,000 robust (|r| > 0.4) and statistically significant (*P*-value < 0.01) correlations with higher |r| were incorporated into network analyses.

Network visualization, modular analysis and node-level topological properties (i.e., degree, betweenness, and closeness centralities) were made with Gephi version 0.9.1. Modules are sub-units or communities, which are sets of highly inter-connected nodes, and the rate of intra-module edges is higher than in inter-module ones. Gephi applies a modularity algorithm called the Louvain method, developed by Blondel et al. to identify module in the network [50]. Degree centrality is the number of paths that connect the local node to other nodes (e.g., connections between taxa). Betweenness centrality is the number of shortest paths going through a node (taxon or environmental factor). Closeness centrality is the number of shortest steps required to access all other nodes from a given node (i.e., connections between taxa and/or environmental factors). The real networks were compared with 1000 Erdös-Réyni random networks, which have the identical number of nodes and edges as the real networks, were generated in the igraph R package [45], with each edge having the same probability of being assigned to any node [51]. Topological characteristics of both real and random networks were calculated and compared, including modularity, clustering coefficient, and average path length.

We follow the widely used approach of Koleff et al. [52] to measure the network dissimilarity between different eukaryotic succession periods, which consists in a re-expression of classical measures of dissimilarity based on a partition of shared and total items [52, 53]:

$$\beta _w = \frac{{a + b + c}}{{\left( {2a + b + c} \right){\mathrm{/}}2}} - 1$$

Where *β*_*w*_ is dissimilarity of networks B and C, a is number of shared edges between networks B and C, b is number of edges unique to network B, c is number of edges unique to network C.

## Results

### Temporal dynamics of cyanobacterial biomass, physical, and chemical factors

We identified one cyanobacterial biomass cycle in both Shidou and Bantou reservoirs that ran from May 2010 to October 2015 (Fig. 1a–d). Cyanobacterial bloomed with almost persistent high biomass from May to September 2010. Although cyanobacterial biomass was still high in late 2010, their biomass rapidly decreased from September 2010 to February 2011, and cyanobacteria showed non-bloom in December 2010 and January 2011. Following this, cyanobacterial biomass was persistently low from March 2011 to July 2014 (except the sample in October 2012 of Bantou Reservoir). However, the cyanobacterial biomass increased from October 2014 to October 2015 and dominated the phytoplankton communities again (Fig. 1a–d). In this study, *Cylindrospermopsis raciborskii* (mean relative biomass was 65.5% of cyanobacteria), *Raphidiopsis* sp. (11.9%), and *Pseudanabaena* sp. (6.8%) dominated the cyanobacteria in Shidou Reservoir, while *C*. *raciborskii* (75.4%), *Raphidiopsis* sp. (9.7%), and *Microcystis flos-aquae* (2.2%) dominated the cyanobacteria in Bantou Reservoir.

The physical and chemical factors showed almost synchronous temporal tendencies in both reservoirs, and correlated with the dynamics of cyanobacterial biomass (Fig. S2). Electric conductivity, pH, and TP decreased from 2010 to 2011 and then increased gradually, whereas transparency, NH_4_-N and NO_x_-N increased from 2010 to 2011 and then decreased gradually in both reservoirs.

The temporal variation in Euclidean distance of environmental factors (cyanobacterial biomass, physical, and chemical factors) showed a strong correlation with the variation in Bray–Curtis dissimilarity of eukaryotic plankton community (Mantel test: Shidou, r = 0.562, *P* < 0.01; Bantou, r = 0.466, *P* < 0.01).

### Temporal dynamics of eukaryotic plankton community composition

The eukaryotic plankton community composition exhibited pronounced and synchronous inter-annual patterns in both Shidou and Bantou reservoirs from May 2010 to October 2015 (Fig. 1e–f). Four distinct eukaryotic succession periods were found according to the NMDS ordinations. Eukaryotic succession period 1—from May 2010 to September 2010, eukaryotic succession period 2—from October 2010 to July 2011, eukaryotic succession period 3—from October 2011 to July 2014, eukaryotic succession period 4—from October 2014 to October 2015. Our clustering results also indicated that (at similarity level of 43.46%) all samples, except a replicate in December 2010 near the inflow, were grouped into four temporal periods corresponding with the NMDS ordination in Shidou Reservoir. However, a replicate in October 2011 near the inflow was grouped into eukaryotic succession period 2 in Shidou Reservoir. All samples (at similarity of 44.67%) were grouped into four temporal periods corresponding with the NMDS in Bantou Reservoir. However, a replicate in September 2010 near the outflow was grouped into eukaryotic succession period 2 in Bantou Reservoir. ANOSIM results indicated that these eukaryotic plankton community compositions in four temporal periods were significantly and clearly separated (Fig. 1e, f, Table 1, global R = 0.709 for Shidou, *P* < 0.01; global R = 0.790 for Bantou, *P* < 0.01). Crucially, the cyanobacteria-based separation was much stronger than seasonal separation in both reservoirs (Table 1).

**Table 1:** Pairwise comparison of eukaryotic plankton communities in Shidou and Bantou reservoirs based on one-way ANOSIM test

Factors	Global R	
	Shidou	Bantou
Inter-annual		
Total (four periods)	0.709**	0.790**
Period 1 vs. period 2	0.903**	0.767**
Period 1 vs. period 3	0.921**	0.963**
Period 1 vs. period 4	0.886**	0.823**
Period 2 vs. period 3	0.525**	0.719**
Period 2 vs. period 4	0.729**	0.742**
Period 3 vs. period 4	0.623**	0.768**
Seasonal		
Four seasons	0.227**	0.157**
Win. & spr. vs. Sum. & aut.	0.185**	0.118**

The ANOSIM statistic R is calculated by the difference of the between-group and within-group mean rank similarities, thus it displays the degree of separation between groups. Complete separation is indicated by R = 1, whereas R =  0 suggests no separation. Total indicates four successional periods. Period 1—eukaryotic community period 1, Period 2—eukaryotic community period 2, Period 3—eukaryotic community period 3, Period 4—eukaryotic community period 4 (see Fig. 1 for more detail). Four seasons indicate comparison among winter, spring, summer, and autumn. *win,* winter (Dec, Jan, and Feb); *spr,* spring (Mar, Apr, and May); *sum,* summer (Jun, Jul, and Aug); *aut,* autumn (Sep, Oct, and Nov)

**P < 0.01

Metazoa accounted for 40.4% of total eukaryotic reads and their relative abundance increased with decreasing cyanobacterial biomass. Most metazoan reads were affiliated to *Eudiaptomus* (the mean relative abundance was 30.3% of eukaryotic plankton—indeed copepods could be visually very abundant in the water samples). The eukaryotic phytoplankton accounted for 24.0% of total eukaryotic reads and their relative abundance decreased with decreasing cyanobacterial biomass. Cryptophyta was the most abundant eukaryotic phytoplankton (6.7%), followed by Chrysophyceae (5.2%), Chlorophyta (4.9%), and Dinophyta (4.4%). Ciliate reads accounted for 5.0 % of total eukaryotic reads (Fig. S3).

### General patterns of eukaryotic species richness and alpha-diversity

The observed total number of eukaryotic OTUs was 8448 in Shidou and 8802 in Bantou reservoirs, respectively. The mean OTU richness was 1101 ± 27 (mean ± s.e.) and 1279 ± 28 in Shidou and Bantou reservoirs, respectively. The mean Shannon–Wiener index was 3.79 ± 0.10 in Shidou Reservoir and 4.20 ± 0.08 in Bantou Reservoir, respectively (Fig. S4).

Both OTU richness and Shannon–Wiener diversity decreased from eukaryotic succession period 1 (bloom) to period 2, followed by an increase from eukaryotic succession period 2 to period 3, and almost unchanged stability from eukaryotic succession period 3 to period 4 (Fig. S4).

### Factors that associated with dynamics of eukaryotic plankton community composition

**Fig. 2:**
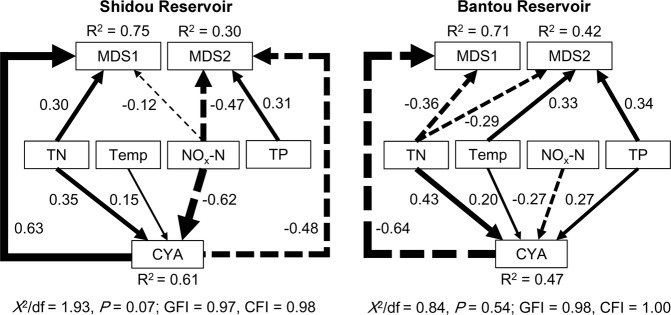
Final path with structural equation model (SEM) showing the relationship between the environmental factors and the first two axes of microeukaryotic MDS ordination in Shidou and Bantou reservoirs. The significant value for the Chi-square test is given, assessing whether the model is significantly different from the corresponding correlation matrix. The goodness-of-fit index (GFI), and Bentler comparative fit index (CFI) indicate the goodness-of-fit of the model. The best fit would result in a value of 1. Numbers on each arrow indicate partial correlation coefficients associated with each causal relationship, and arrow thickness is also proportional to the partial correlation value. Solid line indicates positive correlation, while dash line indicates negative correlation. The determinate coefficient (R^2^) indicates the fraction of the variance that is explained by the model. MDS 1 and MDS 2 represent eukaryotic plankton community NMDS ordination axes 1 and 2, respectively (see Fig. 1), CYA - cyanobacterial absolute biomass (mg/L)

We developed a structural equation model to investigate the linkages between eukaryotic plankton community composition and cyanobacterial biomass cycle (Fig. 2). The GFI (0.97 for Shidou and 0.98 for Bantou), CFI (0.98 for Shidou and 1.00 for Bantou), and *x*^*2*^/df (*x*^*2*^/df = 1.93, *P* = 0.07 for Shidou and *x*^*2*^/df = 0.84, *P* = 0.54 for Bantou) of the trimmed model indicated a good fit of the models to the original data.

In all pathways of both models, the physical and chemical factors significantly correlated with the cyanobacterial absolute biomass (mg/L), and then indirectly influenced the eukaryotic plankton community composition (Fig. 2). In total, the pathways coefficients which correlated with the cyanobacteria accounted for 66.2 and 51.3% of the total pathways coefficients in Shidou and Bantou reservoirs, respectively. All the five physical and chemical factors and cyanobacterial biomass explained 75 and 30% variation of eukaryotic plankton MDS axes 1 and 2 in Shidou Reservoir, respectively. The environmental factors explained 71 and 42% variation of eukaryotic plankton MDS axes 1 and 2 in Bantou Reservoir, respectively.

Mantel tests showed both the cyanobacterial absolute and relative biomass were the strongest factors correlated with the temporal dynamics of the eukaryotic plankton community compared to other physical and chemical factors in both Shidou and Bantou reservoirs (Table S1).

To gain a further understanding of the linkages between different eukaryotic succession periods and cyanobacterial biomass cycle, we explored the characteristics of cyanobacterial biomass in different eukaryotic succession periods. The four eukaryotic succession periods had distinct community composition, and associated with distinct characteristics in cyanobacterial biomass, physical and chemical factors. For example, the eukaryotic succession periods 1 and 3 had persistent bloom and non-bloom, respectively. However, the eukaryotic succession periods 2 and 4 included obviously decreases and increases in cyanobacterial biomass, respectively.

Both mean absolute biomass and mean relative biomass of cyanobacteria were significant different between the four eukaryotic periods (Table S2). The environmental factors (cyanobacterial biomass, physical, and chemical factors) based on the Euclidean distance in these four eukaryotic periods were significantly and clearly separated in both reservoirs (Table S3). During the eukaryotic periods 1 and 3 the cyanobacterial biomass showed weaker correlations with the eukaryotic plankton community. However, in the eukaryotic periods 2 and 4 cyanobacteria exhibited a stronger correlation with the eukaryotic plankton community dynamics (Table S1).

### Factors that associated with eukaryotic plankton co-occurrence networks

**Fig. 3:**
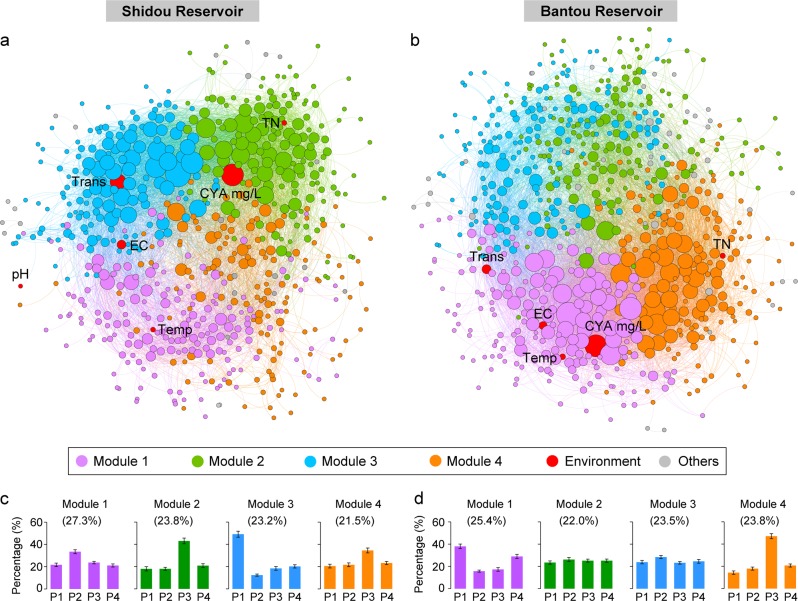
Networks analysis revealing the modular associations between microeukaryotic OTUs and environmental factors in Shidou (**a**) and Bantou (**b**) integrated networks. Relative abundance of microeukaryotic plankton OTUs from major modules in the four different eukaryotic succession periods of Shidou (**c**) and Bantou (**d**) reservoirs. A connection stands for a strong (SparCC |r| > 0.4) and significant (*P*-value < 0.01) correlation. The size of each microeukaryotic OTU or environmental factor (node) is proportional to the number of connections (i.e., degree centrality). Temp, water temperature; EC, electric conductivity; Trans, transparency; TN, total nitrogen; CYA mg/L, cyanobacterial absolute biomass (mg/L); Others, other modules

**Fig. 4:**
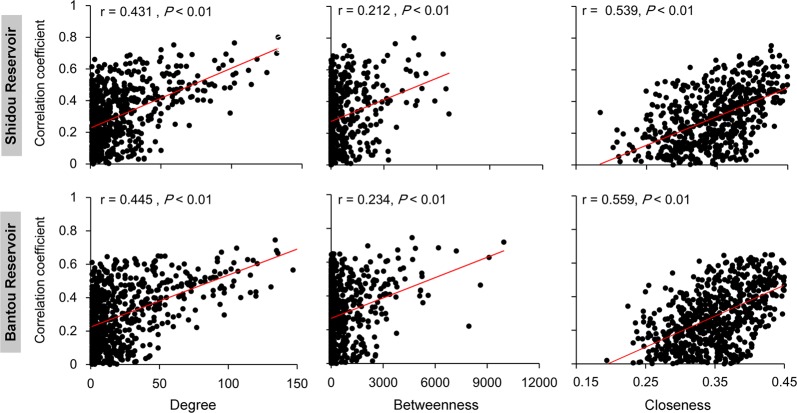
Spearman’s correlations showing the significant relationship between the centrality of microeukaryotic OTUs in the integrated network and their spearman’s correlation coefficients with cyanobacterial absolute biomass (mg/L) in Shidou and Bantou reservoirs

For the integrated networks in Shidou and Bantou reservoirs, cyanobacterial biomass had highest degree, betweenness, and closeness centralities compared to other environmental factors (Fig. 3a, b). This suggests that cyanobacteria in these two reservoirs had the strongest relationships with eukaryotic plankton OTUs compared to other environmental factors. We found only about 20% eukaryotic OTUs (Shidou: 153/633 OTUs, Bantou: 142/701 OTUs) had direct and strong correlations with the cyanobacteria in both Shidou and Bantou integrated networks. However, the top 10 OTUs with the highest degree centrality directly were connected to cyanobacterial biomass in both reservoirs (Fig. S5). Moreover, the eukaryotic plankton OTUs with higher degree, betweenness, and closeness centralities exhibited closer correlations with the cyanobacterial absolute biomass (mg/L) in both the two integrated networks (Fig. 4).

In addition, the properties of two integrated networks and eight sub-networks are summarized in Table S4. All networks had a much higher number of strong positive correlations observed than negative ones, and the percentages of positive correlations were always higher than 60%. The integrated network degrees were distributed according to a power-law distribution in both Shidou and Bantou reservoirs, indicating a scale-free distribution and non-random co-occurrence pattern (Fig. S6). For the integrated networks, although the richness of conditionally rare and abundant taxa was low (Shidou: 138 (21.8% of total OTUs in the Shidou integrated network) OTUs; Bantou: 126 (18.0%) OTUs), they widely associated with rare taxa (Shidou: 44.70%; Bantou: 39.40%) and interrelated with themselves (Shidou: 19.32%; Bantou: 10.46%) (Table S5 and Fig. S7).

We further explored the linkages between eukaryotic plankton co-occurrence networks and different eukaryotic plankton succession periods that associated with the different characteristics in cyanobacterial biomass. The integrated networks in Shidou and Bantou reservoirs clearly parsed into four major modules which accounted for 95.8 and 94.7% of the whole networks, respectively (Fig. 3). These modules were specific to each of four eukaryotic plankton succession periods (Fig. 3c, d). For example, modules II and IV in Shidou Reservoir as well as module IV in Bantou Reservoir were specific to the eukaryotic plankton succession period 3 (non-bloom phase). Module I in Shidou Reservoir and module III in Bantou Reservoir were specific to the eukaryotic period 2 (cyanobacteria decrease phase). Module III in Shidou and module I in Bantou reservoirs were specific to the eukaryotic period 1 (cyanobacteria persistent bloom phase).

Chlorophyta, Ciliophora, Dinophyta, and Stramenopiles had higher degree centrality (interactions with other taxa) in the four major modules of integrated networks in both Shidou and Bantou reservoirs. In particular, the degree centrality of Dinophyta was especially high in modules that corresponding to eukaryotic plankton succession period 1 (bloom state)  in both reservoirs. The degree centrality of Chlorophyta was higher in modules that corresponding to eukaryotic succession period 3 (non-bloom state) in both reservoirs. However, in modules that corresponding to eukaryotic period 2, the Ciliophora and Chrysophyceae had higher degree centrality (Table S6).

The structures of eukaryotic plankton sub-networks in the different eukaryotic succession periods for both reservoirs were not static but varied through time. About 44.0–60.6% eukaryotic OTUs were shared between different sub-networks, but only 2.8–6.3% of correlations shared between different sub-networks (Table 2). More interestingly, this variation of correlations was significantly correlated with the Euclidean distance of environmental factors. The Euclidean distance of cyanobacterial absolute biomass showed the strongest Spearman’s correlation with the dissimilarity of sub-networks (r = 0.756, *P* < 0.01) between different eukaryotic plankton succession periods of Shidou Reservoir, followed by the transparency (r = 0.589, *P* < 0.01) and cyanobacterial relative biomass (r = 0.444, *P* < 0.01). The Euclidean distance of total nitrogen showed the strongest Spearman’s correlation with the variation of sub-networks (r = 0.312, *P* < 0.01) between different eukaryotic plankton succession periods of Bantou Reservoir, followed by the cyanobacterial relative biomass (r = 0.284, *P* < 0.01) and absolute biomass (r = 0.279, *P* < 0.01) (Table S7).

**Table 2:** Number and proportion of shared OTUs and their significant correlations between different sub-networks based on the different eukaryotic plankton succession periods in Shidou and Bantou reservoirs

	OTUs (%)		Correlations (%)
Shidou			
P1–P2	548 (44.0%)	548 (51.2%)	441 (4.4%)
P1–P3	554 (44.5%)	554 (46.6%)	283 (2.8%)
P1–P4	587 (47.1%)	587 (52.1%)	280 (2.8%)
P2–P3	639 (59.7%)	639 (53.7%)	521 (5.2%)
P2–P4	564 (52.7%)	564 (50.0%)	459 (4.6%)
P3–P4	651 (54.8%)	651 (57.8%)	444 (4.4%)
Bantou			
P1–P2	708 (54.2%)	708 (56.8%)	625 (6.3%)
P1–P3	649 (49.7%)	649 (47.2%)	375 (3.8%)
P1–P4	620 (47.4%)	620 (55.1%)	459 (4.6%)
P2–P3	756 (60.6%)	756 (55.0%)	494 (4.9%)
P2–P4	611 (49.0%)	611 (54.3%)	452 (4.5%)
P3–P4	658 (47.9%)	658 (58.4%)	483 (4.8%)

P1—eukaryotic community period 1, P2—eukaryotic community period 2, P3—eukaryotic community period 3, P4—eukaryotic community period 4 (see Fig. 1 for more detail)

Given that the total number of OTUs in different sub-networks is different, two proportions of OTUs are also provided for better comparison. For example, 44.0% is the ratio of shared OTUs to total OTUs in network P1, while 51.2% is the ratio of shared OTUs to total OTUs in network P2

## Discussion

### Mechanisms by which the cyanobacterial biomass cycle is associated with the succession of the eukaryotic plankton community

Although, the relationship between cyanobacterial blooms and bacterial community has been frequently investigated in previous studies [17, 54], few studies have investigated relationship between cyanobacterial blooms and eukaryotic plankton community based on the high-throughput sequencing. A study of this type is necessarily correlative, however these correlations and suggestions about mechanism are based on the known ecology of the organisms involved.

Our results indicated that the cyanobacterial biomass cycle in both Shidou and Bantou reservoirs was strongly correlated with the temporal variation of eukaryotic plankton community composition compared to other physical and chemical factors (Fig. 2, Table S1). However, the physical and chemical factors in the reservoirs, such as water temperature and nutrients were significantly correlated with the cyanobacteria biomass in our structural equation models, and likely indirectly influenced the eukaryotic community composition through their effect on cyanobacteria—as well as by any direct effects (Fig. 2). Previous studies found that increase of water temperatures would favor the growth of cyanobacteria over other plankton species [55]. Water transparency was also found having a strong negative connection to cyanobacteria [8, 56], presumably because blooms reduce light penetration through the water. As some cyanobacteria such as *Cylindrospermopsis raciborskii* can fix N_2_ in their terminal heterocyst cells [56], this allows this species to use systems low in dissolved nitrogen. Consistent with this, Figueredo et al. found this cyanobacterium was favored during periods of low N in a small tropical reservoir [57]. Indeed, in this study we found cyanobacterial biomass (*C*. *raciborskii* overwhelmingly dominated the cyanobacteria biomass in these two reservoirs) had positive correlations with the water temperature, and had negative correlations with the transparency and NO_x_-N in the Shidou and Bantou reservoirs. In addition, the influence of cyanobacterial bloom on protists via effects on heterotrophic bacteria should be considered. Protistan grazing on bacteria is one of the most important ecological processes in microbial food webs that transfer carbon and energy to higher trophic levels [58]. A cyanobacterial bloom can both inhibit (via the release of toxic production) or promote (via the supply of dissolved organic carbon) growth of certain bacteria causing a sequential change in the abundance of heterotrophic nanoflagellates and ciliates [12, 13].

The composition of the eukaryotic plankton community during our study (May 2010–October 2015) was divided into four main clusters (four eukaryotic succession periods) that associated with the four different characteristics of cyanobacterial biomass in the one cyanobacterial biomass cycle (Fig. 1, Table S2). In eukaryotic succession period 1, the cyanobacteria bloom showed persistent high biomass. However, the cyanobacteria steeply declined at the beginning of eukaryotic period 2, and the cyanobacterial biomass changed from bloom to non-bloom in this period. We also found more broken cyanobacterial cells and smaller colonies in this period than in others suggesting that cyanobacteria rapidly senesced during this period. In eukaryotic period 3, cyanobacteria were in non-bloom state with persistent low biomass, except October 2012 in Bantou. In eukaryotic period 4, the cyanobacterial biomass obviously increased from non-bloom to bloom states. During different eukaryotic succession periods, the cyanobacteria showed different correlation strength with the composition of eukaryotic plankton community (Table S1). For example, the cyanobacteria showed stronger correlations with the composition of the eukaryotic plankton community in the eukaryotic periods 2 and 4 than in the eukaryotic periods 1 and 3. The most likely reason is that the cyanobacteria biomass obviously decreased and increased in the eukaryotic periods 2 and 4, respectively. The quick shift in cyanobacterial biomass might associate with quick shift in zooplankton grazing rate on algae [10], heterotrophic bacterial abundance and composition [59, 60], toxins and dissolved organic matters release from algae [6, 61].

### Mechanisms by which the cyanobacterial biomass cycle could affect the eukaryotic plankton co-occurrence network

By analyzing the network, we found the cyanobacterial biomass cycle was strongly linked to eukaryotes in the eukaryotic plankton co-occurrence networks. First, the cyanobacterial biomass had remarkable high centralities in both Shidou and Bantou integrated networks. They exhibited highest degree and betweenness centralities in the Shidou integrated network and had the forth degree and fifth betweenness centralities in the Bantou integrated network (e.g. Fig. 3). Second, although about 20% eukaryotic plankton OTUs (Shidou: 153/633 OTUs, Bantou: 142/701 OTUs) had direct and strong correlations with cyanobacteria in both Shidou and Bantou reservoirs, the top 10 highest degree centrality OTUs directly connected to cyanobacterial biomass in both reservoirs (Fig. S5). Third, we found the eukaryotic plankton OTUs with higher degree, betweenness, and closeness centralities exhibited stronger correlations with the cyanobacterial biomass (Fig. 4).

We further explored the linkages between eukaryotic co-occurrence networks and different eukaryotic succession periods that associated with the different characteristics in cyanobacterial biomass. Several modules were found in both the Shidou and Bantou integrated networks. These modules well corresponded to the four eukaryotic succession periods, and therefore correlated the different characteristics of cyanobacterial biomass in a cyanobacterial biomass cycle (Fig. 3c, d). For instance, modules II and IV in Shidou Reservoir as well as module IV in Bantou Reservoir were specific to the eukaryotic period 3 (non-bloom). Interestingly, the correlations associated with Chlorophyta were greater in these modules (Table S6). Chlorophyta frequently dominated the phytoplankton communities when cyanobacteria were at low numbers, providing evidence for the existence of distinct ecological niches over temporal scales in the reservoir ecosystem in tandem with the disappearance of cyanobacterial bloom [62]. Module I in Shidou Reservoir and module III in Bantou Reservoir were specific to the eukaryotic period 2 (characterized by an obvious cyanobacteria decline) and were found increased correlations associated with Ciliophora (Table S6). The breakdown of cyanobacterial biomass could boost the rate of bacterial growth and production, and heterotrophic Ciliophora are known to be important consumers of both picophytoplankton and bacteria [59]. Therefore, the increase in the correlations of the phylum Ciliophora is most likely derived from the increase in the abundance of their prey. Module III in Shidou and module I in Bantou reservoirs were specific to the eukaryotic period 1 (cyanobacteria persistent bloom) and were found increased correlations associating with Dinophyta (Table S6), especially the genera *Suessiales*, *Prorocentrum*, and *Peridinium*. Fisher et al., in a study of European lakes, suggested that the “ability of Dinophyta to migrate vertically and to supplement their nutrient requirements through heterotrophy may enable them to be at least as successful as cyanobacteria in high nutrient lakes” [63].

Moreover, we constructed four sub-networks for each reservoir based on four eukaryotic community succession periods. We found most eukaryotic plankton OTUs were shared between sub-networks in different eukaryotic periods, but only small parts of significant correlations were shared (Table 2). This suggests that the construction (especially the correlations between OTUs) of sub-networks in different eukaryotic periods of both Shidou and Bantou reservoirs were not constant but varied through time. Furthermore, this variation of network was significantly and strongly correlated with Euclidean distance of cyanobacterial biomass (Table S7). Therefore, these results suggest that associations between the reservoir eukaryotic plankton are not static but varied; this contradicts a common assumption of ecological research [64]. We assume that same OTUs may have diverse co-occurrence patterns, which depend on the local environmental fluctuation. These fluctuations in network structure could be driven by a number of mechanisms acting independently or together; these include time-varying physiological responses, fluctuations in species diversity, and multiple function agents [22].

## Conclusions

In this study, we found one cyanobacterial biomass cycle during 6 years in two subtropical reservoirs—the cyanobacterial bloom (middle 2010), rapid cyanobacteria decrease (late 2010–early 2011), non-bloom (2011–2014), cyanobacteria increase, and second bloom (late 2014–2015). This cycle was strongly correlated with the community composition and co-occurrence networks of eukaryotic plankton. Moreover, the composition of the eukaryotic plankton community was divided into four distinct periods, and eukaryotic plankton co-occurrence in networks also reflected these four periods. The inter-annual variation of eukaryotic plankton community was significantly greater than variation between four seasons from 2010 to 2015. This result may be due to the different characteristics of cyanobacterial biomass in each eukaryotic plankton succession period from a cyanobacterial biomass cycle. Our results also indicated that the co-occurrence patterns of eukaryotic plankton were not static, but they changed over time, thereby highlighting that eukaryotic plankton correlations strongly depend on the environmental fluctuation caused by cyanobacterial blooms. As such the cyanobacteria are operating as ecological engineers [65] altering the environment of the other organisms.

## Supplementary Information

Supplementary Information
