# Supplementary material for: Response of the eukaryotic plankton community to the cyanobacterial biomass cycle over 6 years in two subtropical reservoirs
Source: ISME J. 2019 May 3;13(9):2196–208. doi: 10.1038/s41396-019-0417-9 (PMC6776060; doi:10.1038/s41396-019-0417-9)
Supplement: Supplementary file 1 — Supplementary Information [file 41396_2019_417_MOESM1_ESM.pdf]

Journal: The ISME Journal

Supplementary information of the article:

Response of the eukaryotic plankton community to the cyanobacterial biomass cycle over 6 years in two subtropical reservoirs

Lemian Liu[1,2], Huihuang Chen[1], Min Liu[1,3], Jun R. Yang[1], Peng Xiao[1], David M. Wilkinson[4], Jun Yang[1,*]

[1] *Aquatic EcoHealth Group, Key Laboratory of Urban Environment and Health, Institute of Urban Environment, Chinese Academy of Sciences, Xiamen 361021, China*

[2] *Technical Innovation Service Platform for High Value and High Quality Utilization of Marine Organism, Fuzhou University, Fuzhou 350108, China*

[3] *University of Chinese Academy of Sciences, Beijing 100049, China*

[4] *School of Life Sciences, University of Lincoln, Lincoln LN6 7TS, UK.*

Running title: Eukaryotic plankton linked to cyanobacterial biomass

***Corresponding author:**

E-mail address: jyang@iue.ac.cn (Jun Yang); Tel. / Fax: +86-592-6190775.

This supplementary information contains:

- 16 Pages
- 7 Figures
- 7 Tables
- 1 Reference

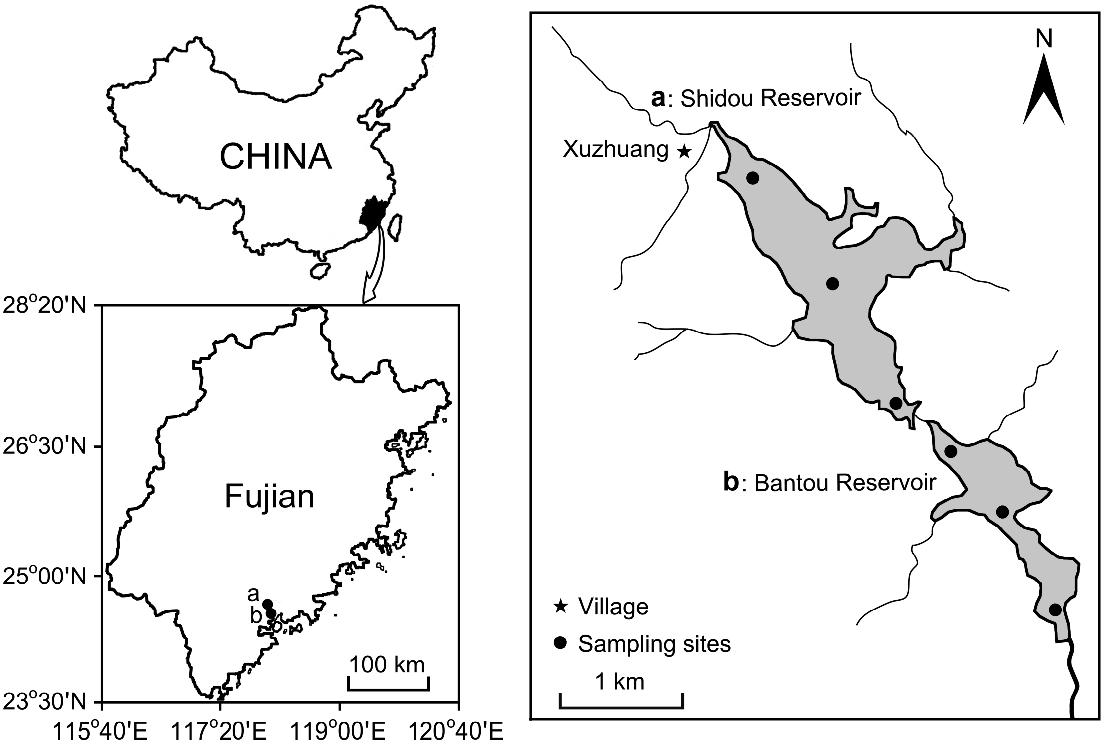

Fig. S1 Map showing the Shidou and Bantou reservoirs and sampling sites in Fujian province from subtropical China (revised from Yang *et al*., 2017).

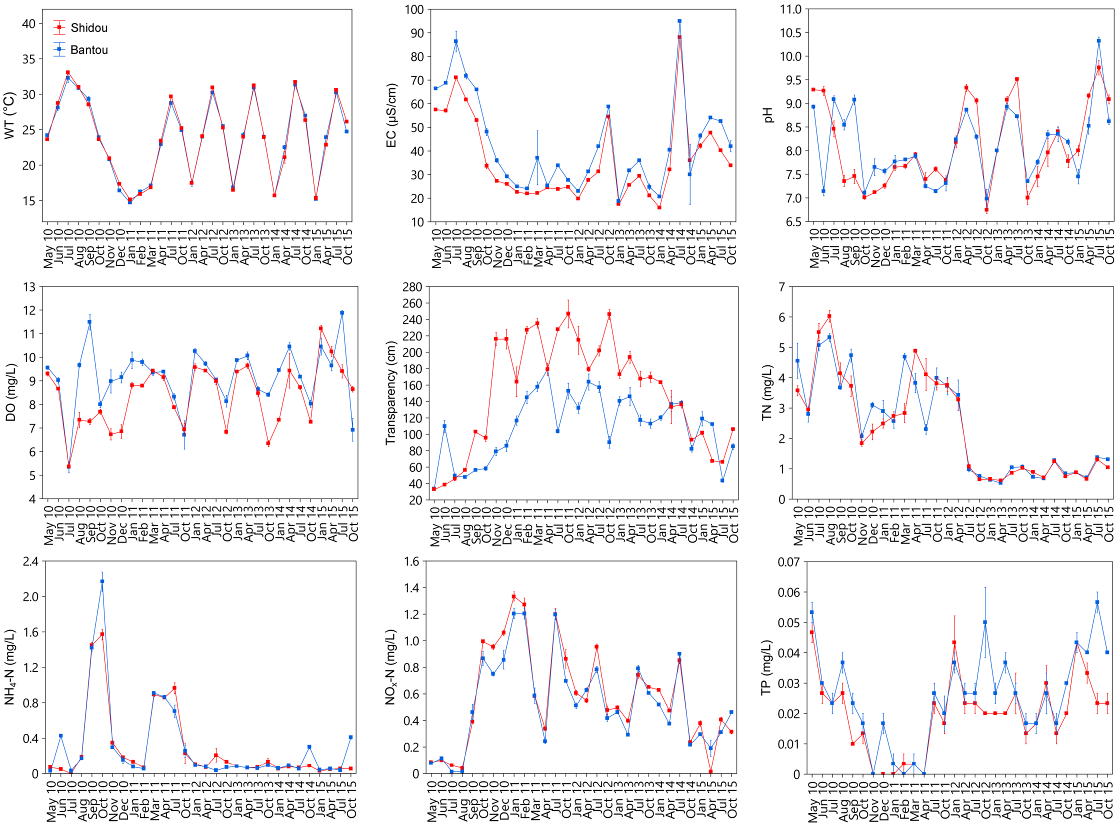

Fig. S2 Variation of 9 environmental parameters in Shidou and Bantou reservoirs from May 2010 to October 2015. WT - water temperature, EC - electrical conductivity, DO - dissolved oxygen, TN - total nitrogen, TP - total phosphorus. Error bars represent standard error of the mean.

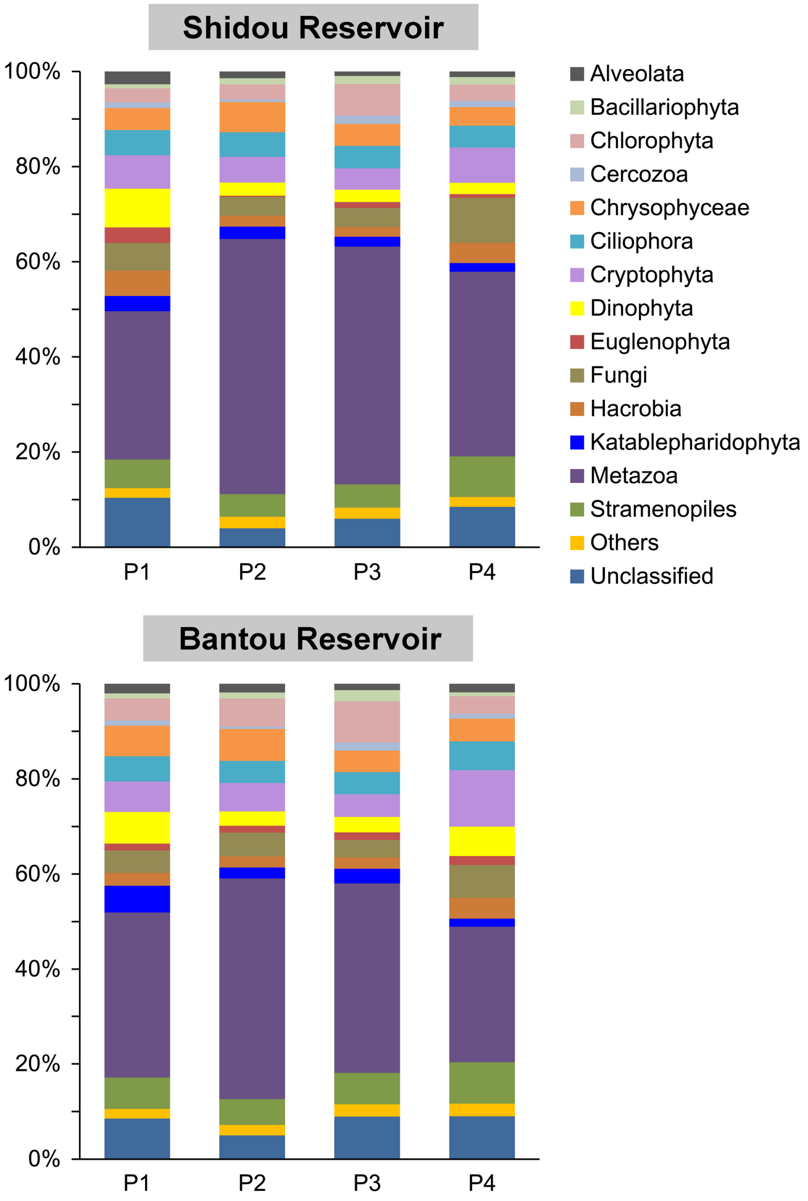

Fig. S3 Relative abundance of microeukaryotic plankton based on Illumina high-throughput sequencing in Shidou and Bantou reservoirs from May 2010 to October 2015. Phyla with mean relative abundances < 1% were merged together as "Others". P1 - eukaryotic succession period 1, P2 - eukaryotic succession period 2, P3 - eukaryotic succession period 3, P4 - eukaryotic succession period 4 (see Fig. 1 for more detail).

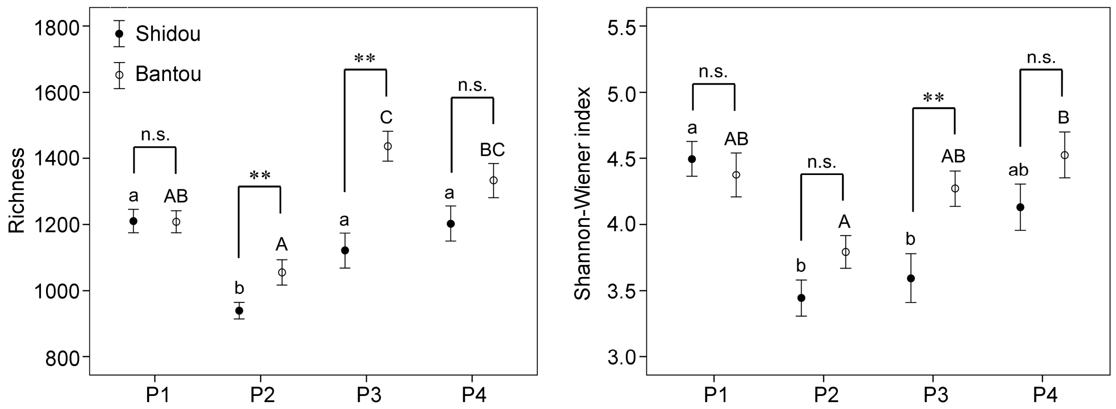

Fig. S4 Variation of the microeukaryotic OTU richness and the Shannon-Wiener index in Shidou and Bantou reservoirs from May 2010 to October 2015. Different lowercase and capital letters indicate the significant difference at 5% level in Shidou and Bantou reservoirs, respectively. Statistical analysis is one-way ANOVA. P1 - eukaryotic succession period 1, P2 - eukaryotic succession period 2, P3 - eukaryotic succession period 3, P4 - eukaryotic succession period 4 (see Fig. 1 for more detail).

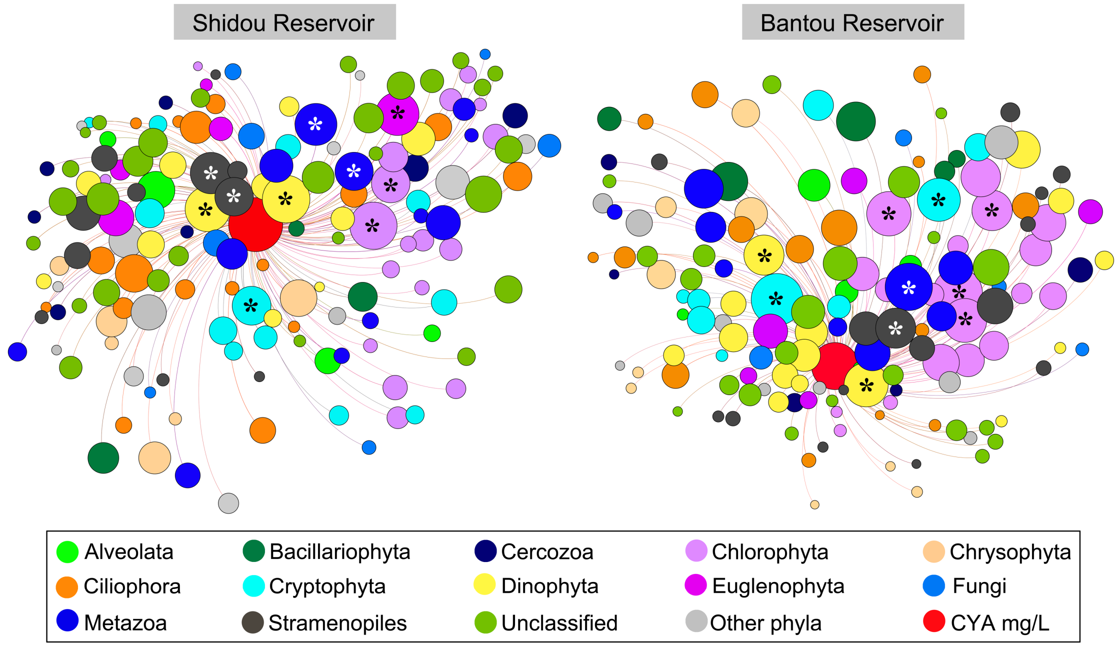

Fig. S5 Network between cyanobacteria biomass and its directly connected OTUs from the integrated networks in Shidou and Bantou reservoirs, respectively. Each node represents a microeukaryotic OTU or cyanobacterial absolute biomass. Node size is proportional to node degree. Star (*) marked nodes were the top 10 OTUs with the highest degree. CYA mg/L - cyanobacterial absolute biomass (mg/L).

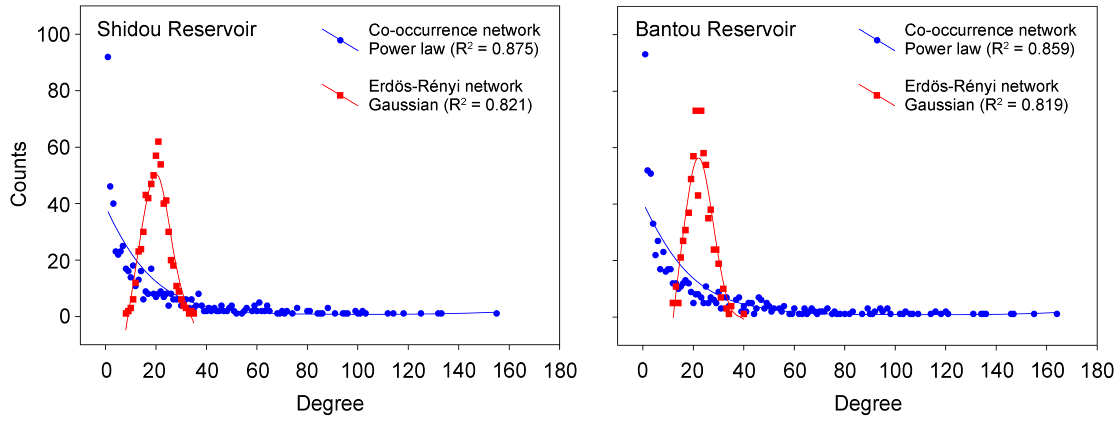

Fig. S6 The distributions of degree for the eukaryotic plankton integrated networks (blue) and Erdös-Rényi random networks (red) in Shidou and Bantou reservoirs.

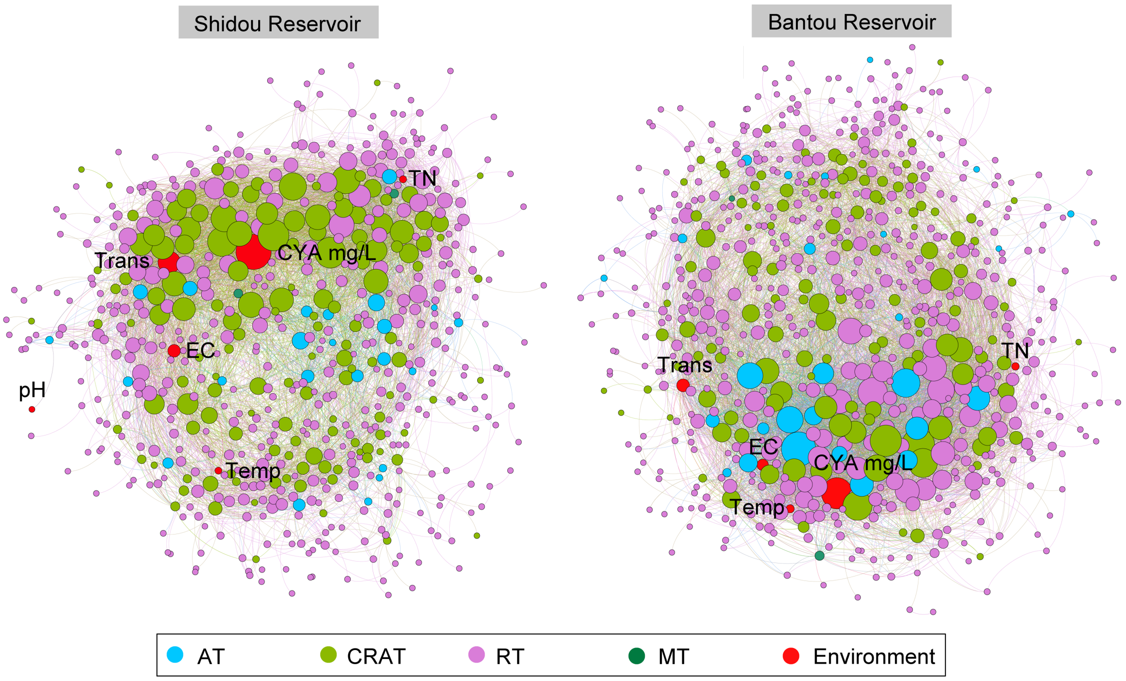

Fig. S7 Networks analysis revealing the associations among abundant taxa (AT), conditionally rare and abundant taxa (CRAT), rare taxa (RT), moderate taxa (MT) of microeukaryotes, and environmental factors in Shidou and Bantou integrated networks. Each node represents a microeukaryotic OTU or an environmental factor. A connection stands for a strong (SparCC's $|r| > 0.4$) and significant (P-value < 0.01) correlation. The size of each node is proportional to the number of connections (i.e., degree). Temp - water temperature, EC - electric conductivity, Trans - transparency, TN - total nitrogen, CYA mg/L - cyanobacterial absolute biomass (mg/L).

Table S1 Mantel tests for the correlation between community similarity and environmental factors using Spearman's coefficient

	Shidou Reservoir					Bantou Reservoir				
	Total	P1	P2	P3	P4	Total	P1	P2	P3	P4
Temp	0.205**	0.304**	0.116	0.168**	**0.539****	0.221**	0.242*	0.259**	**0.455****	0.436**
EC	0.412**	0.366**	0.494**	**0.270****	0.283**	0.375**	0.095	0.379**	**0.408****	0.142
pH	0.164**	0.564**	0.423**	0.170**	0.339**	0.195**	0.192*	0.461**	-0.003	0.516**
DO	0.095*	0.325**	**0.519****	0.170**	0.313**	0.160**	**0.295****	0.181*	0.079	0.332**
Trans	**0.481****	**0.732****	0.133	**0.251****	0.381**	**0.397****	0.118	**0.475****	-0.025	0.608**
TN	0.268**	0.286**	0.158*	0.124*	0.334**	0.280**	-0.111	0.071	0.113	**0.649****
TP	0.119**	0.382**	0.000	-0.001	0.333**	0.180**	**0.279****	0.248**	-0.083	0.214*
NH_4-N	0.051	**0.637****	0.181*	0.079	0.129	0.110*	**0.271****	0.202*	0.076	0.056
NO_x-N	0.327**	**0.635****	0.243**	0.158*	0.146	0.280**	0.091	0.110	**0.175****	0.062
CYA (mg/L)	**0.574****	0.422**	**0.624****	**0.367****	**0.386****	**0.478****	0.029	**0.540****	0.028	**0.717****
CYA (%)	**0.464****	0.632**	**0.590****	0.091	**0.400****	**0.445****	-0.065	**0.559****	-0.002	**0.612****

* $P < 0.05$, ** $P < 0.01$

Bold fonts indicate the top three highest values

P1 – eukaryotic succession period 1, P2 – eukaryotic succession period 2, P3 – eukaryotic succession period 3, P4 – eukaryotic succession period 4 (see Fig. 1 for more detail)

Temp - water temperature, EC - electrical conductivity, DO - dissolved oxygen, Trans - water transparency, TN - total nitrogen, NH_4-N - ammonium nitrogen, NO_x-N - nitrite and nitrate nitrogen, TP - total phosphorus

CYA (mg/L) - cyanobacterial absolute biomass (mg/L), CYA (%) - percentage of cyanobacterial biomass to total algae (%)

Table S2 Cyanobacterial absolute biomass and relative biomass (the percentage of cyanobacterial biomass to total algae, %) in Shidou and Bantou reservoirs

	Shidou Reservoir		Bantou Reservoir	
	Biomass (mg/L)	Biomass (%)	Biomass (mg/L)	Biomass (%)
P1	2416.9 ± 332.3	97.1 ± 1.3%	74.0 ± 10.7	79.4% ± 5.7%
P2	12.6 ± 4.6	22.9 ± 6.4%	6.7 ± 1.6	34.3 ± 4.3%
P3	0.4 ± 0.1	6.6 ± 0.7%	2.7 ± 1.2	12.0 ± 2.8%
P4	42.3 ± 6.5	81.9 ± 2.6%	22.0 ± 5.9	55.0 ± 7.8%

P1 – eukaryotic succession period 1, P2 – eukaryotic succession period 2, P3 – eukaryotic succession period 3, P4 – eukaryotic succession period 4 (see Fig. 1 for more detail)

Table S3 Pairwise comparison of environmental variables in Shidou and Bantou reservoirs based on one-way ANOSIM test

Factors	Global R	
	Shidou Reservoir	Bantou Reservoir
**Inter-annual**		
Total (four periods)	0.613**	0.509**
Period 1 vs. Period 2	0.918**	0.746**
Period 1 vs. Period 3	0.983**	0.803**
Period 1 vs. Period 4	0.745**	0.284**
Period 2 vs. Period 3	0.210**	0.331**
Period 2 vs. Period 4	0.700**	0.481**
Period 3 vs. Period 4	0.620**	0.417**
**Seasonal**		
Four seasons	0.218**	0.184**
Win. & spr. vs. Sum. & aut.	0.076**	0.124**

** $P < 0.01$

The environmental variables consist of 11 parameters, namely water temperature, electric conductivity, pH, dissolved oxygen, transparency, total nitrogen, NH_4-N, NO_x-N, total phosphorus, cyanobacterial absolute biomass (mg/L) and percentage of cyanobacterial biomass to the total algae (%)

The ANOSIM statistic R is calculated by the difference of the between-group and within-group mean rank similarities, thus it displays the degree of separation between groups. Complete separation is indicated by R = 1, whereas R = 0 suggests no separation

Total indicates four successional periods of eukaryotic plankton community. Period 1 – eukaryotic succession period 1, Period 2 – eukaryotic succession period 2, Period 3 – eukaryotic succession period 3, Period 4 – eukaryotic succession period 4 (see Fig. 1 for more detail)

Four seasons indicate comparison among winter, spring, summer and autumn. Win. – winter (Dec, Jan and Feb), Spr. – spring (Mar, Apr and May), Sum. – summer (Jun, Jul and Aug), Aut. – autumn (Sep, Oct and Nov)

Table S4 Properties of the integrated networks in eukaryotic plankton from Shidou and Bantou reservoirs

	Nodes	Edges	Positive edges (%)	Negative edges	Average betweenness
Shidou integrated	639	6465	4178 (64.6%)	2287 (35.4%)	616.1
Shidou P1	1246	10000	6367 (63.7%)	3633 (36.3%)	1729.6
Shidou P2	1070	10000	6069 (60.7%)	3931 (39.3%)	1312.2
Shidou P3	1189	10000	6073 (60.7%)	3927 (39.3%)	1375.4
Shidou P4	1127	10000	6838 (68.4%)	3162 (31.6%)	1459.4
Bantou integrated	706	7936	5014 (63.2%)	2922 (36.8%)	658.7
Bantou P1	1307	10000	6385 (63.9%)	3615 (36.1%)	1718.2
Bantou P2	1247	10000	6345 (63.5%)	3655 (36.5%)	1586.6
Bantou P3	1374	10000	6208 (62.1%)	3792 (37.9%)	1749.4
Bantou P4	1126	10000	6436 (64.4%)	3564 (35.6%)	1450.8

P1 – eukaryotic succession period 1, P2 – eukaryotic succession period 2, P3 – eukaryotic succession period 3, P4 – eukaryotic succession period 4 (see Fig. 1 for more detail)

Table S5 Number of edges and ratio of edges to pairwise correlations between different eukaryotic subgroups and environmental factors

	Shidou Reservoir	Bantou Reservoir
AT-AT	32 (0.49%)	72 (0.91%)
CRAT-CRAT	**1249 (19.32%)**	**830 (10.46%)**
RT-RT	**1424 (22.03%)**	**2381 (30.00%)**
MT-MT	0 (0%)	0 (0%)
AT-CRAT	263 (4.07%)	425 (5.36%)
AT-RT	285 (4.41%)	**838 (10.56%)**
AT-MT	2 (0.03%)	4 (0.05%)
CRAT-RT	**2890 (44.70%)**	**3126 (39.4%)**
CRAT-MT	12 (0.19%)	6 (0.08%)
RT-MT	17 (0.26%)	11 (0.14%)
Environment-Environment	3 (0.05%)	4 (0.05%)
Taxa-Environment	288 (4.45%)	239 (2.99%)

Bold fonts indicate the values > 10%

AT - abundant taxa, CRAT - conditionally rare and abundant taxa, RT - rare taxa, MT - moderate taxa

Table S6 Number of degree of different eukaryotic groups in different modules in Shidou and Bantou integrated networks

	Shidou Reservoir				Bantou Reservoir			
	M1	M2	M3	M4	M1	M2	M3	M4
Alveolata	79	47	161	88	189	**227**	83	101
Bacillariophyta	171	174	24	44	181	152	74	93
Cercozoa	27	136	150	2	111	68	62	164
Chlorophyta	147	**734**	136	**549**	228	141	225	**1730**
Chrysophyceae	**369**	64	239	60	315	123	**444**	87
Ciliophora	**426**	335	475	175	583	193	**518**	226
Cryptophyta	152	57	262	**326**	460	189	130	235
Dinophyta	233	363	**665**	80	**934**	145	**267**	286
Euglenophyta	23	123	233	13	256	0	97	68
Fungi	79	116	241	105	161	192	144	153
Metazoa	149	**510**	220	134	**713**	80	67	253
Stramenopiles	**267**	75	**591**	**192**	464	**332**	213	**424**
Others	193	229	164	35	182	42	200	225
Unclassified	174	**946**	**661**	130	**826**	**281**	124	**944**

M1 - module I, M2 - module II, M3 - module III, M4 - module IV

Bold fonts indicate the top three highest values

Others - other phyla, Unclassified - unclassified eukaryotes (sequence similarity >80%)

Table S7 Spearman's correlation between the dissimilarity of sub-networks and Euclidean distance of environmental factors in different eukaryotic plankton succession periods of Shidou and Bantou reservoirs

	Shidou Reservoir	Bantou Reservoir
Water temperature	-0.035	-0.145**
Electrical conductivity	0.433**	0.113**
pH	-0.015	-0.034
Dissolved oxygen	0.123**	0.149**
Water transparency	**0.589****	0.205**
Total nitrogen	0.143**	**0.312****
Total phosphorus	-0.180**	-0.116**
NH_4-N	-0.196**	-0.129**
NO_x-N	0.157**	0.015
CYA (mg/L)	**0.756****	**0.279****
CYA (%)	**0.444****	**0.284****

** $P < 0.01$

Bold fonts indicate the top three highest Spearman's coefficients (absolute value)

P1 – eukaryotic succession period 1, P2 – eukaryotic succession period 2, P3 – eukaryotic succession period 3, P4 – eukaryotic succession period 4 (see Fig. 1 for more detail)

CYA (mg/L) - cyanobacterial absolute biomass, CYA (%) - percentage of cyanobacterial biomass to total algae

Reference

Yang JR, Lv H, Isabwe A, Liu LM, Yu XQ, Chen HH, Yang J. Disturbance-induced phytoplankton regime shifts and recovery of cyanobacteria dominance in two subtropical reservoirs. ***Water Res***. 2017; 120: 52–63.
